# Can They See It? The Functional Field of View Is Narrower in Individuals with Autism Spectrum Disorder

**DOI:** 10.1371/journal.pone.0133237

**Published:** 2015-07-23

**Authors:** Yongning Song, Yuji Hakoda, Wakako Sanefuji, Chen Cheng

**Affiliations:** 1 Key Laboratory of Brain Functional Genomics, Ministry of Education, Shanghai Key Laboratory of Brain Functional Genomics, School of Psychology and Cognitive Science, East China Normal University, Shanghai, China; 2 Faculty of Human Development and Education, Kyoto Women's University, Kyoto, Japan; 3 Faculty of Human-Environment Studies, Kyushu University, Fukuoka, Japan; 4 Department of Psychology, University of Massachusetts Boston, Boston, Massachusetts, United States of America; Hamamatsu University School of Medicine, JAPAN

## Abstract

Although social cognitive deficits have long been thought to underlie the characteristic and pervasive difficulties with social interaction observed in individuals with autism spectrum disorder (ASD), several recent behavioral and neuroimaging studies have indicated that visual perceptual impairments might also play a role. People with ASD show a robust bias towards detailed information at the expense of global information, although the mechanisms that underlie this phenomenon remain elusive. To address this issue, we investigated the functional field of view in a group of high-functioning children with autism (n = 13) and a paired non-ASD group (n = 13). Our results indicate that the ability to correctly detect and identify stimuli sharply decreases with greater eccentricity from the fovea in people with ASD. Accordingly, a probe analysis revealed that the functional field of view in the ASD group was only about 6.62° of retinal eccentricity, compared with 8.57° in typically developing children. Thus, children with ASD appear to have a narrower functional field of view. These results challenge the conventional hypothesis that the deficit in global processing in individuals with ASD is solely due to weak central coherence. Alternatively, our data suggest that a narrower functional field of view may also contribute to this bias.

## Introduction

Recently, the nonsocial symptoms of autism spectrum disorder (ASD) have been the subject of increasing focus. The idea that individuals with ASD perceive the world differently, for instance, in cases of superior performance on perceptual tasks, is perhaps the most intriguing of all of the puzzles associated with autism (see [1]). For example, individuals with autism may exhibit superior performance, or show intact ability, on tasks where a local processing bias is beneficial, such as the Block Design task [2], [3], the Embedded Figures Test (EFT) [4], [6], the Navon task [7–10], the drawing tasks [11–12], and on visual search task [13–16]. Further evidence that people with ASD assign an increased level of attention to local visual information has emerged from the results of a counting experiment [17], in which children with autism tended to count dots individually, rather than enumerating them (see also [18]).

Why do individuals with ASD demonstrate a perceptual preference for local information or an impairment of global information processing? One cognitive theory for this local bias proposes that individuals with autism exhibit weak central coherence (WCC), reflecting a deficit in the central control processes that are responsible for drawing together component features into a coherent whole (see [19], [20]). In contrast, typically developing individuals have a natural tendency to automatically perceive stimuli as a whole by combining individual elements. Happé and Frith published an updated review of the WCC literature (2006) [21]. Although it is reasonable to expect that a weak coherence mechanism might underlie poor performance on visuospatial tasks in children with autism, some researchers have indicated that the observed local bias may not be entirely due to WCC. For example, several studies failed to demonstrate a superior local bias in people with autism for tasks expected to favor an emphasis on local processing (e.g., [22], [23]). Furthermore, several reports found that local processing is enhanced in individuals with autism, but not at the expense of global processing [24], [25].

Although the transformation of perceptual information into a central coherence signal is important for processing global information, we argue that strong central coherence is not the only effective cognitive explanation for the precedence of global processing in individuals without ASD. For example, in terms of the processing of compound stimuli, previous studies have revealed that many factors can facilitate or impair the tendency to use global aspects versus local elements (e.g., the Navon pattern) (see [26]). These factors include 1) the sparsity with which the local elements are placed across the visual field, 2) the visual angle subtended by the compound stimulus, and 3) the location of the compound stimulus within the visual field relative to the point of fixation (see [27]). Together, these results suggest that humans ‘zoom-out’ to respond to global stimuli and ‘zoom-in’ to respond to local stimuli. Additionally, Lamb and Robertson (1988) demonstrated that peripheral presentations lead to global precedence, whereas central presentations do not [28]. Specifically, they found that the human visual system depends more on the peripheral visual field when processing global information compared with local information, meaning that stimuli presented further from the fovea on the retina will elicit more processing of global versus local information.

If global processing is highly dependent on peripheral vision, then it is possible that poor performance on global processing tasks exhibited by individuals with ASD is not only caused by WCC, but also by poor peripheral visual perception. However, no study has systematically questioned how gradual changes in the degree of retinal eccentricity would affect visual perception in the two participant groups. Thus, to assess whether individuals with ASD differ from controls in terms of visual perception, it is necessary to measure the functional field of view (FFoV) using technology which permits a precise investigation of acuity in terms of degree of eccentricity from the fovea in individuals with ASD.

Although the definition of the FFoV has been somewhat controversial [29–31], most researchers define the FFoV as an area of the visual field (mainly encompassed by the peripheral vision) within which a target can be perceived. If the FFoV is narrower in people with ASD, this may explain both the observed deficits associated with encoding the global aspects of a stimulus and the superior local processing performance exhibited by such individuals. It is important to note that the WCC theory and the narrower FFoV theory are two hypotheses that do not conflict. The former postulates that individuals with ASD can see all stimuli but fail to make an appropriate integration (i.e., pull together individual elements to perceive stimuli as Gestalts). The latter postulates that individuals with ASD cannot see stimuli presented far away from the fixation point. Consequently, they cannot further process these stimuli, and so they fixate their attention on the central visual field. Both hypotheses offer a reasonable explanation of the local processing bias in people with ASD.

In the current study, we assessed the FFoV of participants with ASD and age-matched controls to investigate the cognitive mechanisms underlying the local bias seen in people with ASD. In the FFoV task, digits appeared on a screen at different degrees of retinal eccentricity, and participants were asked to detect and identify these digits. By testing whether individuals with ASD and controls performed differently on the FFoV task, we hoped to elucidate the source of the local processing bias seen in individuals with ASD.

## Methods

### Ethics statement

All procedures were approved by the internal review board of Kyushu University, and written informed consent was obtained from all participants and their parents prior to testing.

### Participants

Children diagnosed with high-functioning autism or Asperger’s syndrome (HFA/AS) were recruited from Shanghai Pediatric Hospital. Before testing, we obtained written consent from the participants and their parents. Each family completed an unstructured screening interview based on the Child and Adolescent Psychiatric Assessment [32]. We recorded information regarding the medical history, developmental history, and general symptoms of all participants. We excluded children with any significant comorbid psychiatric or neurological conditions, such as epilepsy, severe attention deficit hyperactivity disorder, or schizophrenia.

All children in the autism group met the criteria specified in the *DSM-IV-TR* [33]. Parents of children with ASD filled out the Asperger Syndrome (and high-functioning autism) Diagnostic Interview (ASDI) [34], the Childhood Asperger Syndrome Test (CAST) [35], and the Autism Spectrum Screening Questionnaire (ASSQ) [36]. All of the individuals with ASD received a score in the autistic range (screening cutoff was 15 or above) on the ASDI and a score indicating minimal to mild autism on the CAST (6 or above). Furthermore, we administered Raven’s Progressive Matrices IQ test [37] to all participants. Children exhibiting intellectual disabilities (IQ scores below 85) were excluded from further analysis.

Thirteen children with ASD (*Mean age* = 11.46, *S*.*D*. = 2.60) and normal visual acuity were included in the ASD group. The control group comprised children recruited from primary schools. The control group included 13 children with normal visual acuity (*Mean age* = 11.38, *S*.*D*. = 1.76) who received a score of less than seven (as scored by their parents) on the ASSQ [32] (screening cutoff was 7 or below e.g., [38]). Children from the control group were matched with children in the ASD group based on age and nonverbal mental ability. We found no significant differences between groups in terms of performance on Raven’s Progressive Matrices IQ test (ASD: *Mean* = 94.46, *S*.*D*. = 9.5; non-ASD: *Mean* = 98.0, *S*.*D*. = 12.2; *t* (24) = −0.824, *p* = 0.42) (see Table 1).

**Table 1:** Demographic and symptom variables of participants.

ASD	1	2	3	4	5	6	7	8	9	10	11	12	13
Type	AS	AS	HFA	HFA	AS	HFA	HFA	HFA	HFA	HFA	HFA	AS	AS
Sex	M	F	M	M	M	M	M	M	M	M	M	M	M
Age	10	8	8	14	14	8	10	10	13	14	13	15	12
IQ (RPM)	121	91	102	89	90	104	92	90	93	89	89	90	88
ASDI	25	23	27	16	21	17	23	18	22	29	24	25	27
CAST	13	15	10	14	14	9	8	9	10	13	16	12	12
CONTROL	1	2	3	4	5	6	7	8	9	10	11	12	13
Sex	M	M	M	M	M	M	M	M	M	M	M	M	M
Age	14	14	14	12	12	9	9	11	10	11	11	11	10
IQ (RPM)	105	108	120	90	88	91	92	90	92	89	94	124	92
ASSQ	1	1	1	0	3	1	0	0	2	2	1	0	1

Note. ASD: Autism Spectrum Disorder. AS: Asperger Syndrome. HFA:High-Functioning Autism. M: Male. F: Femal. RPM: Raven’s Progressive Matrices. ASDI: the Asperger Syndrome (and high-functioning autism) Diagnostic Interview. CAST: the Childhood Asperger Syndrome Test. ASSQ: the Autism Spectrum Screening Questionnaire.

### Apparatus and stimuli

We conducted our experiments using the Psychtoolbox extension in Matlab (MathWorks, Inc., Natick, MA) [39, 40]. We used a personal computer (DELL PRECISION M6800) to generate stimuli and collect data. One of four digits (1, 3, 4, 7), which subtended a visual angle of 0.5° vertically and 0.5° horizontally, was selected randomly and presented on a gamma-corrected 17.3-inch Mobile PC Display, which subtended a visual angle of 35.6°×20.8° Stimulus presentation was synchronized with the monitor’s vertical refresh rate (75 Hz).

To capture the real-time fixation position of their eyes, we simultaneously recorded the eye movements of each participant. Eye movements were recorded at a sampling rate of 60 Hz with the Tobii X2-60 eye-tracker (Tobii Technology), which has an average gaze position error of 0.5° and a near-linear output over the range of the monitor used. We conducted a manual calibration of two-eye fixations at the beginning of each session using a five-point fixation procedure implemented via Tobii Studio software. We performed drift correction for each trial.

### Procedure and design

**Fig 1:**
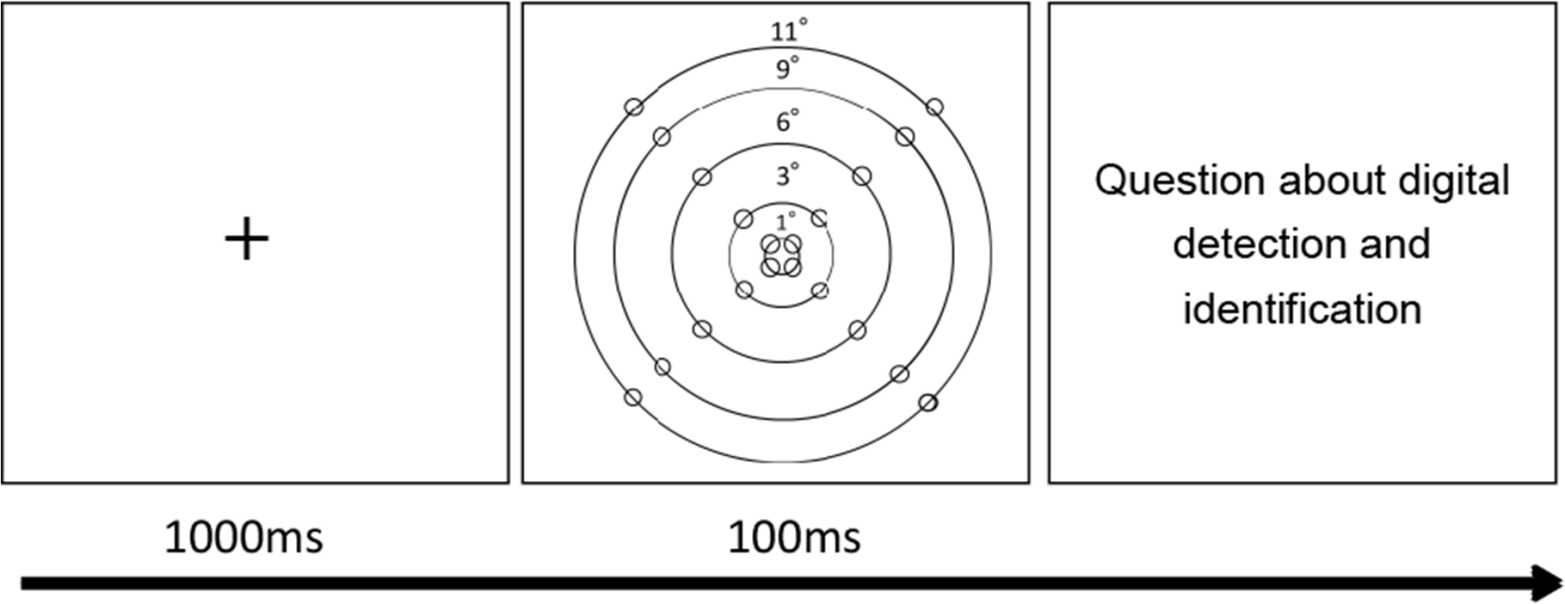
Schematic representation of the sequence of experimental events. The onset of the stimulus could be triggered by pressing the space key. After displaying a fixation stimulus for 1000 ms, one digit was randomly presented in one of 20 positions (as represented by the figure with dots) within five degrees of eccentricity (i.e., 1°, 3°, 6°, 9°, and 11°) for 100 ms. The participants then answered questions about stimulus detection and identification. Note that the lines, dots, and degree numbers in the second panel of this figure were not present in the actual test stimuli.

Fig 1 shows the sequence of events in each experimental trial. Initially, the participants were asked to fixate binocularly on a central cross. Each trial began with a 1000-ms fixation period, followed by the presentation of each stimulus for 100 ms. We recorded the real-time gaze point of the eyes in each trial and set five conditions for the degree of retinal eccentricity (i.e., 1°, 3°, 6°, 9°, and 11°).

The degree of retinal eccentricity represents how far a given point in the visual field is located from the fixation point (fovea). For example, a retinal eccentricity value of 9° means that the target (i.e., digit in the current experiment) is located 9° away from the real fixation point. In our experiment, each digit could appear in one of 20 randomly varied positions within the five retinal eccentricity conditions. Six trials contained a blank stimulus to test whether the participants’ responses were arbitrary.

The participants were asked to use a keyboard to respond to questions displayed on the screen. In the first question, they were asked whether they had noticed a digit. If the participant indicated “yes,” they were then asked the following question: “What was the digit?” To respond to this question, participants selected one of five alternatives (1, 3, 4, 7, or “I do not know”). The stimulus presentation ended when the participant answered the second question; a response immediately triggered the next trial. Before testing began, the participants were fully informed about the length of the experiment and the response requirements. Prior to initiating the real experiment, we conducted 12 practice trials. The participants who could not follow the instructions (e.g., appeared to be guessing their answers) or who were unable to cooperate were excluded from further experimentation. Each participant completed 90 trials in the actual test: there were six conditions (blank trial, 1°, 3°, 6°, 9°, and 11°) presented 15 times each.

### Data analysis

First, we identified outlier data associated with eye blinks and off fixation trials (approximately 2% of all trials), and removed these from further analysis. Second, we calculated the ratio of correct detection, i.e., the number of correctly detected stimuli to the total number of stimuli presented in each degree condition of the digital detection task. We also calculated the ratio of correct identification, i.e., the number of correctly identified stimuli to the total number of stimuli presented in each degree condition of the digital identification task. We performed two-way analyses of variance (ANOVAs) to analyze the differences between the two groups for these parameters. Third, we performed a probit analysis for the ratio of correct identification. The probit method, invented by Fechner (1947), is a process by which a sigmoid response curve is generated from original data by transforming the responses based on a normal integral [41]. It is a method that is commonly used to analyze similar psychophysics experiments. For each observer, a cumulative Gaussian function was fitted to the ratio of correct identification according to a maximum-likelihood method [42].

At this point, we did not conduct any further eye tracking analysis. We used eye tracking solely to capture the real-time fixation position of the eyes with respect to the five degrees of retinal eccentricity conditions.

## Results

### Ratio of correct detection

We performed a two-way ANOVA on the ratio of correct detection with group (ASD or non-ASD) as the between-subjects factor and degree of eccentricity as the within-subjects factor. We found that the main effect of group type was significant (*F (1*, *24) = 19*.*82*, *p < 0*.*001*), and that the main effect of eccentricity was statistically significant (*F (4*, *96) = 7*.*75*, *p < 0*.*001*). Additionally, the interaction effect between group type and the degree of eccentricity was significant (*F (4*, *96) = 6*.*22*, *p < 0*.*001*).

**Fig 2:**
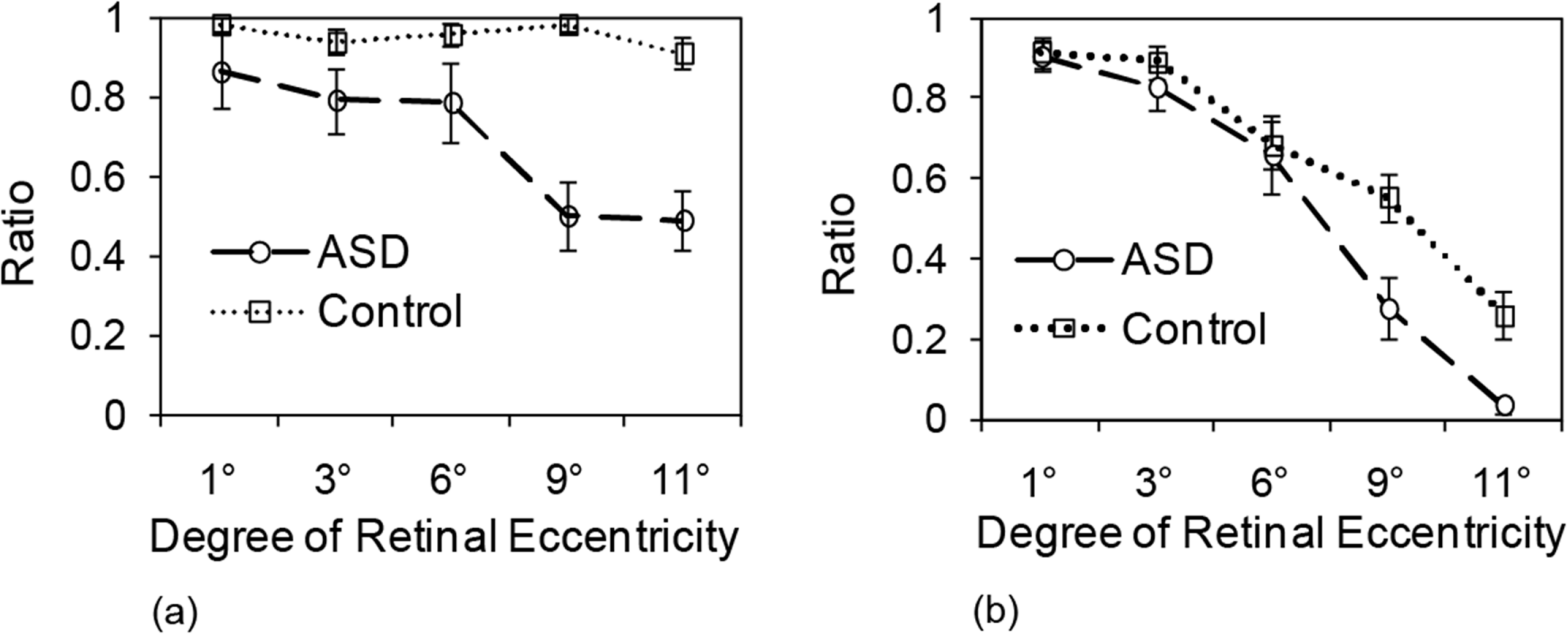
Ratios of correct detection of digits. Mean ratio of correct detection of digit (a) and mean ratio of correct identification of digit (b). Error bars: Standard Errors.

Further analysis of the simple main effects revealed that group was only significant for 6°, 9°, and 11°(*F* (1, 120) = 4.03, *p* = 0.05; *F* (1, 120) = 31.47, *p* < 0.001; *F* (1, 120) = 24.37, *p* = 0.001) and was not significant for 1°and 3° (*F* (1, 120) = 1.95, *p* = 0.17; *F* (1, 120) = 0.09, *p* = 0.09) (Fig 2A).

### Ratio of correct identification

We performed a two-way ANOVA on the ratio of correct identification with group (ASD or non-ASD) as a between-subjects factor and degree of eccentricity as a within-subjects factor. We found that the main effect of group type was significant (*F (1*, *24) = 11*.*69*, *p < 0*.*01*), and the main effect of eccentricity was also significant (*F (4*, *96) = 71*.*07*, *p < 0*.*001*). The interaction effect between group type and the degree of eccentricity was significant (*F (4*, *96) = 2*.*61*, *p = 0*.*04*).

Further analysis of the simple main effects revealed that group was only significant for 9°and 11° (*F* (1, 120) = 13.33, *p* < 0.01; *F* (1, 120) = 8.44, *p* < 0.01) and was not significant for 1°, 3°, and 6° (*F* (1, 120) = 0.02, *p* = 0.89; *F* (1, 120) = 0.67, *p* = 0.42; *F* (1, 120) = 0.10, *p* = 0.76) (Fig 2B).

### Probit analysis of the correct identification ratios

**Fig 3:**
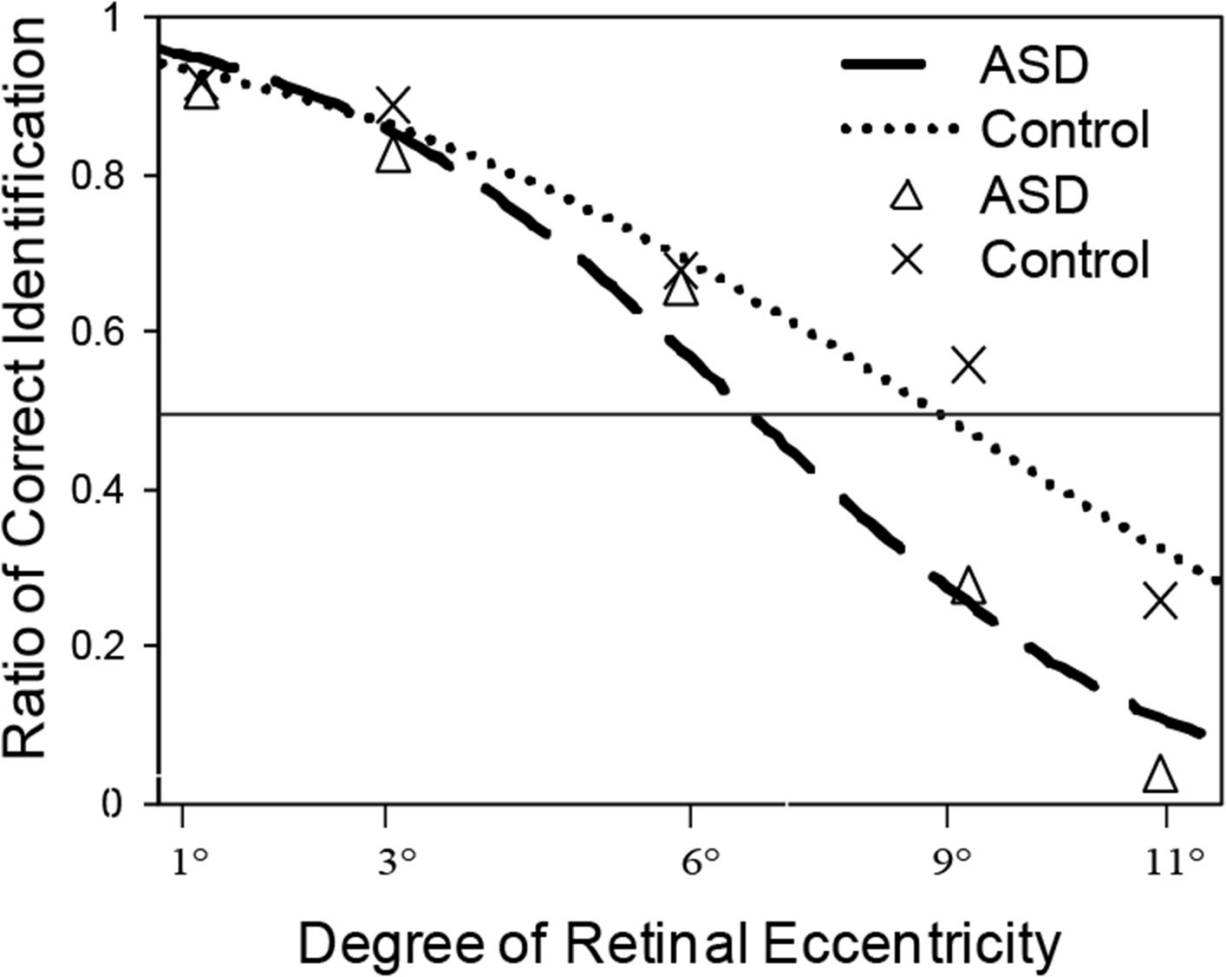
Predictions for the ratio of correct identification at different degrees of eccentricity. Dashed lines represent fitted cumulative Gaussian functions; horizontal solid lines represent ‘ambiguous’ percepts (correct identification ratio equals 50%). The degree of retinal eccentricity when the correct identification ratio equaled 50% was considered to be the operational definition for the functional field of view for the two groups.

Fig 3 shows the means across observers as a function of the degree of retinal eccentricity, along with the best-fit lines for the pooled data. The best-fit lines were qualitatively consistent with the means of the best-fit data from individual observers. As the degree of retinal eccentricity increased, individuals with ASD exhibited a sharp decrease in acuity for digit identification. However, the values corresponding to the functional field of view for the two groups were still unclear. To address this, we defined the functional field of view in the current study by taking the degree at which 50 percent of attempted identifications were correct (i.e., the degree of ambiguous percepts in the field of view). Based on the best-fit lines, we estimated the degree of retinal eccentricity where the ratio of correct identification would be 0.5 for each group. We then did an independent sample t-test to determine whether these numbers were significantly different between groups (6.62° and 8.57°). We found that the effect was significant (*t* (24) = 2.39, *p* < 0.05) (Fig 3).

## Discussion

The goal of this study was to investigate the cognitive mechanisms underlying poor global processing in people with autism. Using an eye tracking method, we measured the FFoV in a group of children with ASD and a group of typically developing children. We found that the correct detection ratio and the correct identification ratio decreased more sharply with greater eccentricity from the fovea in participants with ASD compared with the control group. The FFoV was narrower in the ASD group than in the control group (6.62° for the ASD and 8.57° for the control group). These findings are in agreement with earlier studies showing that visual attention in individuals with autism is spatially overfocused [27], [43].

To the best of our knowledge, the specific finding that people with ASD have narrower FFoVs has not been reported previously. These results challenge the conventional hypothesis that the poor performance on global information processing or bias towards details in individuals with ASD is solely due to WCC [19–20]. The narrower FFoV could also explain the superior local bias behaviors exhibited by people with ASD when completing the Block Design subtest of the Wechsler intelligence scales [5] and the Embedded Figures Task [5], [6]. In these cases, the narrower FFoV (i.e., a sharp falloff in acuity) would imply the reduced impact of the global aspects of a stimulus and the increased impact of the local aspects of a stimulus. However, these findings do not completely refute the WCC hypothesis in people with ASD. This is because observers were not required to integrate information across different parts of the visual field in the current study. Because the WCC and FFoV hypotheses are not entirely in conflict with one another, it is still possible that people with autism have deficits associated with integrating visual stimuli and extracting meaning from a visual display.

And the findings of the current study also related to superior visual search in autism. Although it has long been postulated that the visual search superiority in ASD derived from enhanced perception of stimulus features or enhanced ability to discriminate between targets and distractors at the locus of attention [44], the FFoV might also play a role. For example, it has been thought that the visual search (i.e., a single feature or a conjunction features) requires attention to small elements [45]. The narrower FFoV facilitates the attention to small features, thus the visual search improves in ASD. If this is the case, then we expected that during the visual search, eye movement would be impaired in autism. Specifically, the spatial distribution of fixations across the search array might be significantly narrower among children with ASD. It is necessary to examine this question in future research.

The narrower FFoVs in people with ASD might also be related to social cognitive deficits (e.g., face perception). Although it has long been postulated that the social cognitive deficits exhibited by people with ASD are associated with pervasive problems with social interaction and/or motivation, several recent studies suggest that a visual perceptual impairment might also contribute. The argument for atypical perception underlying a facial processing impairment is based on the finding that individuals with ASD are particularly local-biased, and concurrently, may fail to extract global information from faces [46]. For example, individuals with ASD showed a reduced or absent “face inversion effect” and a reduced “face composite effect” [47–49], which was considered to be related to the deficit in global processing.

Our findings have important implications for understanding the neural deficits involved in visual processing in people with ASD. Visual signals are converted into electrical signals at the retina and transmitted to the primary visual cortex (V1). In V1, visual signals are represented by a series of neurons that fall within a limited area (i.e., the neuron’s receptive field, as described in detail previously [45]). In primates, visual information from the retina is projected to V1 via two independent but linked pathways- the magnocellular pathway, which has relatively larger receptive fields, and the parvocellular pathway, which has relatively smaller receptive fields [50–52]. Indeed, many recent studies employing visual stimuli designed to selectively stimulate the magnocellular and parvocellular pathways have indicated that ASD may be associated with abnormal magnocellular pathway function [53–55]. If this is the case, then the narrower FFoV found in people with ASD might be due to a deficit in the magnocellular pathway.

In summary, this study represents a pilot investigation of the FFoV in individuals with ASD. By comparing the FFoV in children with ASD with that of typically developing children, we investigated the cognitive mechanisms underlying the local bias seen in people with ASD. We found that people with ASD exhibited a sharp falloff in acuity with greater eccentricity from the fovea, indicating that these individuals have a narrower FFoV. Our results challenge the conventional hypothesis that the bias towards details in individuals with ASD is solely due to WCC. We suggest that the narrower FFoV may provide an alternate explanation for global processing deficits in people with ASD.

## Supporting Information

S1 Table Ratio of correct identification%. (DOCX)
