# Supplementary material for: Can They See It? The Functional Field of View Is Narrower in Individuals with Autism Spectrum Disorder
Source: PLoS One. 2015 Jul 23;10(7):e0133237. doi: 10.1371/journal.pone.0133237 (PMC4512679; doi:10.1371/journal.pone.0133237)
Supplement: S1 Table — (DOCX) [file pone.0133237.s001.docx]

Supporting Information

**S1 Table. Ratio of correct identification%**

|  |  | 1° | 3° | 6° | 9° | 11° |
| --- | --- | --- | --- | --- | --- | --- |
| 1 |  | 67 | 100 | 50 | 75 | 0 |
| 1 |  | 83 | 67 | 67 | 67 | 15 |
| 1 |  | 100 | 80 | 100 | 25 | 20 |
| 1 | control | 67 | 100 | 67 | 67 | 25 |
| 1 |  | 100 | 100 | 80 | 34 | 60 |
| 1 |  | 100 | 100 | 75 | 25 | 34 |
| 1 |  | 100 | 67 | 34 | 75 | 34 |
| 1 |  | 100 | 100 | 50 | 67 | 0 |
| 1 |  | 100 | 100 | 67 | 67 | 67 |
| 1 |  | 83 | 100 | 100 | 80 | 15 |
| 1 |  | 92 | 90 | 69 | 56 | 26 |
| 1 |  | 100 | 80 | 80 | 34 | 20 |
| 1 |  | 100 | 75 | 50 | 50 | 20 |
| 2 |  | 100 | 100 | 100 | 0 | 0 |
| 2 |  | 75 | 100 | 67 | 67 | 0 |
| 2 |  | 67 | 33 | 33 | 0 | 14 |
| 2 |  | 100 | 80 | 100 | 0 | 0 |
| 2 | ASD | 100 | 90 | 100 | 20 | 10 |
| 2 |  | 95 | 100 | 47 | 20 | 0 |
| 2 |  | 75 | 75 | 80 | 25 | 0 |
| 2 |  | 100 | 90 | 70 | 0 | 0 |
| 2 |  | 75 | 67 | 0 | 50 | 0 |
| 2 |  | 91 | 83 | 66 | 28 | 4 |
| 2 |  | 100 | 100 | 20 | 75 | 0 |
| 2 |  | 100 | 80 | 75 | 25 | 0 |
| 2 |  | 100 | 80 | 100 | 50 | 20 |
